# Editorial: Racial and ethnic inequalities in multiple long-term conditions: current trends and viable solutions

**DOI:** 10.3389/fpubh.2026.1800730

**Published:** 2026-03-04

**Authors:** Patrick Oyibo, Brenda Hayanga

**Affiliations:** 1 School of Health and Medical Sciences, City St. George's, University of London, London, United Kingdom; 2 Department of Global Health & Social Medicine, Faculty of Social Science & Public Policy, King's College London, London, United Kingdom

**Keywords:** ethnicity, health inequalities, multiple long-term conditions, race, structural determinants

## Introduction

The presence of two or more long-term health conditions [i.e., multiple long-term conditions (MLTCS)] poses significant challenges for individuals and health systems (1). MLTCs are associated with poor quality of life, increased morbidity, mortality (2), healthcare utilization, polypharmacy (3) and a greater need for multi-department consultations (4). The overall global prevalence of MLTCs is estimated at 37.2% (5) and is projected to increase in the coming decades (6). Yet, the prevalence of MLTCs and its impact is not felt equally across racial/ethnic groups. A growing body of evidence suggests that racially/ethnic minoritized people prematurely and disproportionately experience the burden MLTCs (7–9). The Research Topic, *Racial and ethnic inequalities in multiple long-term conditions: current trends and viable solutions*, was conceptualized to showcase the state of the art in racial/ethnic inequalities in MLTCs and illuminate viable solutions to address existing inequalities. In this Editorial, we critically discuss the seven articles contributing to this Research Topic, all of which add to the vast body of literature documenting racial/ethnic inequalities in health(care) outcomes. First, we describe the scope of the studies, and the key themes that cut across the articles. Second, we discuss the ways in which these articles advance our understanding of racial/ethnic inequalities in MLTCs. Lastly, we consider the implications for policy and practice to address existing health inequalities (including MLTCs).

## Overview of contributing articles

Of the seven articles comprising this Research Topic, five articles provide insights into health(care) outcomes and experiences of racially/ethnic minoritized people living in countries such as the UK (*n* = 1) and the US (*n* = 4) which have historically contributed to the established body of literature on racial/ethnic inequalities in health. Two articles provide insights into racial/ethnic inequalities among understudied populations living in China (*n* = 1) (Deng et al.), and Germany (*n* = 1) (Gangarova et al.). Collectively, the articles provide insight into the circumstances and experiences of a range of racial/ethnic minoritized groups including Black, African, Afro-diasporic and/or Muslim healthcare users in Germany (Gangarova et al.); Black/African American, Latino, Asian people in the US (Ramirez et al.; Jimenez et al.; Shafquat et al.; Saiyed et al.); Black, Asian and Mixed ethnic people in the UK (Au-Yeung et al.); and Zhiguo, Akha, Zhuang, Dai, Yi and Hmong ethnic minority groups in China (Deng et al.).

The contributing articles adopt a range of research designs, thereby, providing a comprehensive understanding of racial/ethnic inequalities in health(care). The quantitative studies (*n* = 5) alert us to the characteristics of long-term conditions, MLTCs and their impact on ethnic minority populations in Yunan province in China (Deng et al.), racial inequalities in infant mortality (Saiyed et al.), racial differences in knowledge, attitudes toward vaccination, and risk practices related to Lyme Disease (LD) (Shafquat et al.), the association of socioeconomic deprivation and ethnicity on the risk of diabetes (Au-Yeung et al.), and the disproportionate financial hardship experienced by Black, Latino, and Asian households with children during the COVID-19 pandemic (Jimenez et al.). Conversely, the qualitative studies (*n* = 2) provide in-depth insights into health(care) experiences of racially/ethnic minoritized people (Gangarova et al.) and health providers perspectives of the influences of Cancer disparities among Latinos in the US (Ramirez et al.).

These articles span different conditions, stages of the disease process and aspects of the care continuum. Crucially, the articles give insight into the pathways through which single conditions progress to MLTCs. For instance, Ramirez et al. suggest that the stress of a Cancer diagnosis can also result in mental health issues, underscoring the link between physical and mental health conditions. Similarly, Deng et al. posit that delayed or inadequate diagnosis and treatment of chronic diseases further contribute to rapid disease progression and preventable disability, particularly among vulnerable populations. In their study examining the association of socioeconomic deprivation and ethnicity on the risk of diabetes, Au-Yeung et al. found that socioeconomic deprivation increased the risk of (pre)diabetes. They hypothesize that limited income, lower health literacy, housing instability, and reduced food access negatively influence an individual's ability to effectively manage their health and increases the risk of developing complications associated with diabetes (Au-Yeung et al.).

## Cross cutting themes

Across all articles, a central theme is the interaction between structural and social factors in producing racial and ethnic inequalities in health and healthcare, including the development and management of MLTCs. Ramirez et al. identify a combination of environmental and genetic influences, cultural and linguistic barriers, health behaviors, and systemic access issues as key contributors to Cancer disparities among Latino populations in the US. Others further demonstrate how racially/ethnic minoritized groups are disproportionately concentrated in occupations and living environments that adversely affect health outcomes (Deng et al.; Ramirez et al.; Shafquat et al.). Deng et al., for example, highlight how harsh living conditions and geographical and infrastructural barriers restrict health literacy and self-care capacity, increasing vulnerability to disease. Similarly, Au-Yeung et al. show how structural constraints such as poverty, limited education, and inadequate housing reduce access to healthy food and opportunities for physical activity, thereby, worsening health outcomes and quality of life.

Several studies explicitly position structural racism as an upstream driver of health inequalities (Gangarova et al.; Jimenez et al.; Saiyed et al.). Saiyed et al. link city-level poverty rates with both overall infant mortality rates and Black infant mortality rates. Jimenez et al. demonstrate how systemic racism exacerbated pre-existing economic inequality during the COVID-19 pandemic. Gangarova et al. further reveal the dual mechanism by which structural racism operates. First, healthcare users are discriminated against in healthcare encounters. Second, they avoid healthcare services due to prior experiences of being othered, ignored or unheard (Gangarova et al.). These repeated experiences cultivate mistrust in healthcare systems among racially/ethnic minoritized healthcare users constraining their capacity engage with care (Gangarova et al.).

## Implications for policy and practice

The articles collectively demonstrate how structural processes, particularly racism and discrimination, interact with social factors to shape the conditions in which racial/ethnic minoritized groups live and work, as well as their access to, and use of healthcare services for managing health conditions. Together, the findings highlight important implications for policymakers and practitioners seeking to reduce racial/ethnic health inequalities, including those related to MLTCs. Several contributions emphasize the need for further research to deepen understanding of these inequalities. For example, Shafquat et al. identify racial differences in knowledge, attitudes, and practices related to LD as contributors to inequitable outcomes, calling for further investigation into information sources, trust in those sources, and how these vary by race/ethnicity. Similarly, Saiyed et al. report the lack of statistically significant associations between some structural racism composite indices and inequity measures, underscoring the need to refine or identify different indicators of structural determinants in urban health research.

Other studies recommend tailored/targeted interventions to address inequities (Deng et al.; Au-Yeung et al.), alongside greater attention to inclusion, cultural relevance, and accessibility in both research and care delivery (Ramirez et al.). Echoing this, Saiyed et al. stress the importance of engaging local communities to understand contextual histories, identify priority issues, and co-develop effective interventions. Multiple authors further highlight the need to address upstream structural drivers (e.g., poverty, unemployment, income inequality, and residential segregation) to inform equitable policy responses (Jimenez et al.; Saiyed et al.; Au-Yeung et al.). Finally, Gangarova et al. caution against overlooking racialisation processes, which reveal how subtle, normalized forms of racism are enacted within healthcare systems. They argue for anti-racist policies that move beyond cultural competence to address racism at structural, institutional, and interpersonal levels.
